# Intrathecal baclofen therapy in patients with spastic paraplegia: retrospective evaluation of pretreatment drugs, test dosage, dose increments and final therapy

**DOI:** 10.1016/j.bas.2025.104323

**Published:** 2025-07-05

**Authors:** Stephan Kurz, Linda Maria Marth, Christoph-Heinrich Hoffmann, Jonas Hermann Maria Kummerant, Frederik Wilhelm Schneckmann

**Affiliations:** a Department for Spinal Cord Injuries, BG Unfallklinik Frankfurt am Main, Friedberger Landstraße 430, 60389, Frankfurt, Germany; b Department of Spinal and Neurosurgical Surgery, BG Unfallklinik Frankfurt am Main, Friedberger Landstraße 430, 60389, Frankfurt, Germany

**Keywords:** Intrathecal baclofen therapy, Spastic paraplegia, Spasticity

## Abstract

**Introduction:**

Intrathecal baclofen therapy (ITB) is an effective alternative for severe generalized spasticity after unsuccessful oral spasmolytic therapy.

**Research question:**

What degree of severity, type and duration of pretreatment indicated ITB? What are safe test, starting and effective treatment dose? How common are complications?

**Material and methods:**

Descriptive retrospective analysis. Variables: age, gender, etiology, ASIA Impairment Scale (AIS), neurological level, Ashworth Scale (AS). Time of implantation after onset of paralysis. Baclofen test dose, escalation, effective treatment. Type of complications.

**Results:**

27 patients (21 male, 6 female) were examined. 80 % were classified AIS A. Neurological levels: 55 % cervical, 45 % thoracic. Median onset of paralysis: 12 months prior. 31 % were previously treated with triple, 54 % with double and 15 % monotherapy. In 42 %, 50 μg intrathecal baclofen test dose was sufficient; 34 % required over 100 μg. Mean increase in daily flow rate from implantation was approx. 180 μg. Effective dose averaged 360 μg per day. All patients started at AS IV°, with 40 % improving to AS I° and 60 % to AS II° under ITB. Complications occurred in 25 % (infection, skin perforation, catheter occlusion, pump malfunction). 85 % of them could be further treated intrathecally after revision.

**Discussion and conclusion:**

ITB requires close clinical care and individual coordination depending on the level of pretreatment. Further analysis of complications needs larger case numbers.

## Highlights

• Intrathecal baclofen is effective for severe spinal spasticity after oral therapy failure.
• Indication follows failed combination therapy with oral antispasmodics.
• Individual dose adjustment is crucial; average effective dose is 360 μg/day.
• Complication rate is 25 %, with 85 % continuing therapy after revision.

## 1 Introduction

Spasticity and muscle spasms are prevalent complications following central neurological disorders such as spinal cord injury (SCI) or multiple sclerosis (Wang et al., 2020), affecting up to 80 % of individuals with chronic SCI (Nevin et al., 2017).

The negative consequences of spasticity are varied and may include pain, reduced ability to perform daily life activities, increased risk of developing pressure injuries and reduced quality of life. An upper motor neurone syndrome leading to spasticity can appear by an increase in muscle tone and hyperexcitability of stretch reflexes.

Spontaneous muscle contractions can provoke severe painful sensations and can be triggered by passive movements or cutaneous stimulation as well as environmental impacts.

Likewise, infections, wounds or broken bones can significantly affect the level of spasticity. Depending on the severity, muscle spasms can interfere with ability and activities of daily life, increase the risk of falls from a wheelchair and may also lead to sleep disturbance (McIntyre et al., 2014; Rabchevsky and Kitzman, 2011).

Spinal spasticity typically affects antigravity muscles—flexor muscles in the upper extremities and extensor muscles in the lower extremities (Katz, 1988).

Various therapeutic approaches are available, ranging from nonmedical treatments, such as physiotherapy and occupational therapy, to pharmacological and interventional methods.

Centrally acting oral agents, such as GABA B agonists (e.g., baclofen) and anticonvulsants (e.g., clonazepam), have become standard for reducing spasticity and muscle spasms. However, these systemic antispastic drugs frequently induce undesirable sedative side effects, which can considerably impair quality of life.

GABA B agonists may also lead to muscle weakness and ataxia. Consequently, systemic oral therapy often cannot be titrated to the desired effective dose and must be discontinued when adverse side effects predominate.

This issue is particularly pronounced in cases requiring combination therapy or high medication doses, where unfavorable side effects frequently lead to treatment discontinuation. Moreover, the risk of toxicity and withdrawal syndromes necessitates vigilant monitoring and prompt recognition by clinicians.

Due to its lipophilic nature, oral baclofen has a limited capacity to cross the blood-brain barrier compared to intrathecally administered baclofen. Intrathecal administration achieves higher CNS concentrations at the spinal cord level with lower doses, thereby avoiding many systemic side effects. Intrathecal baclofen is particularly effective for treating lower limb spasticity, as the drug concentration is believed to be highest at lower spinal level (Kischka, 2008). Before proceeding with pump implantation, an intrathecal test screening must be conducted to confirm the efficacy and safety of this administration route for the individual patient. A baclofen trial is essential to evaluate the drug's effectiveness at the spinal level and to assess its safety profile before the permanent implantation of an intrathecal baclofen (ITB) pump (Simon and Yelnik, 2010).

In particular, the episode of oral dose reduction before starting intrathecal testing represents a stressful phase for the patient, as spasticity may initially increase considerably. Furthermore, special in-hospital precautions (including monitoring of vital parameters and possibly treatment in the intensive care unit) must be taken during the first intrathecal test. Since the clinical effect of the intrathecal test should be assessed as clearly as possible to ensure a correct and reliable indication for the implantation of an intrathecal baclofen pump, monitoring of the test must be carefully planned and conducted. Moreover, multiple attempts with increasing test dosages may be necessary to avoid inadvertently provoking acute overdoses. At the same time, the total number of lumbar punctures should be kept as low as possible to minimize the risk of infection. Ultimately, when deciding to implant a medication pump, it is crucial that the starting dosage is selected safely and that the presumed target dosage—which is always very individual and can depend on various influencing factors (e.g., accompanying stress factors, mild urinary tract infections, exposure to cold, etc.)—can be achieved as quickly as possible to suppress the functionally impairing and sometimes painful spasticity in a clinically satisfactory manner, thereby ensuring a safe and successful transition from inadequate oral spasticity treatment with side effects to intrathecal spasticity treatment with, ideally, fewer side effects. Given these factors, this study investigates whether a trend can be identified between the number and dosage of oral antispastic medications used and the effective intrathecal baclofen dose required for clinically significant improvement in spasticity. Since target dosages vary widely for individual patients, investigating this relationship could provide valuable insights into optimal baclofen testing and dosing strategies and, ideally, allow for more individualized, safe, and efficient scheduling of initial intrathecal treatment.

When the potential risks of intrathecal application are carefully managed, this treatment can provide a highly effective alternative for addressing chronic generalized spasticity in patients for whom oral treatments have failed.

## 2 Methods

### 2.1 Study design and data collection

We conducted a retrospective, descriptive study. Data were collected from individuals with both acute and chronic traumatic and non-traumatic spinal cord injury, as well as a small sample of non-SCI patients. Most patients were treated in a specialized SCI center unit. Although some underwent pump implantation in external clinics, subsequent care, including medication reservoir refills, was managed at our hospital. SCI-experienced physicians and therapists carried out data collection. Neurological assessments were conducted in accordance with the International Standards for Neurological Classification of Spinal Cord Injury (ISNCSCI). For the study sample, anonymized data were extracted from a clinical database.

### 2.2 Study population

The inclusion criteria for the study sample were as follows: patients receiving intrathecal baclofen (ITB) treatment for acute or chronic traumatic and non-traumatic spastic spinal cord injury, or patients with neurological disorders causing spasticity or muscle spasms due to upper motor neuron syndrome. Demographic data (age, gender) and characteristics of paralysis, including cause and neurological level of injury (NLI), were documented. The severity of SCI was assessed using the ASIA (American Spinal Injury Association) Impairment Scale (AIS), and all clinical examinations adhered to the ISNCSCI (International Standards for Neurological classsification of Spinal Cord Injury).

### 2.3 Primary and secondary outcomes

The primary outcome measures included the intrathecal baclofen test dose, dose escalation, and the effective treatment dose. Additional parameters included spasticity levels, which were evaluated using the Ashworth Scale (AS) both before and after ITB pump implantation. Other parameters involved the time of pump implantation relative to the onset of paralysis, as well as the type and frequency of complications observed.

### 2.4 Evaluation of prior oral drug management

Another aspect of our study was evaluating the prior oral spasmolytic therapy used by patients before initiating ITB therapy. Typically, patients received oral spasticity pretreatment using baclofen, tizanidine, clonazepam, and dantrolene. We classified patients into three categories based on their previous treatment: monotherapy, dual therapy, or triple therapy. The relationship between prior oral therapy and the required intrathecal baclofen screening dose was assessed, with the goal of minimizing lumbar punctures while establishing the indication for pump-assisted baclofen treatment. This analysis was incorporated into the results section, where we examined how prior therapy influenced the required baclofen doses for effective spasticity control.

### 2.5 Neurological characteristics and clinical assessments

Paralysis characteristics recorded cause and neurological level of injury (NLI). SCI severity was reflected with ASIA Impairment Scale (AIS). All clinical examinations followed the International Standards of Neurological Classification in Spinal Cord Injury (ISNCSCI). The level of spasticity was assessed with the Ashworth Scale (AS) pre and post ITB pump implantation. The total Ashworth score is found by summing grades for hip flexion, hip abduction, knee flexion, and ankle dorsiflexion on each side, then dividing them by eight. The ashworth scales measures resistance to passive stretch with a rating of 0–4, with 0 indicating no increase in tone and 4 indicating that the affected region is rigid in flexion or extension. The AS is a five-point scale (grade 0–4)3.

### 2.6 Statistical analysis

Descriptive statistics (mean, standard deviation, frequency, ratio and percentage) were used for epidemiological and demographic characteristics.

## 3 Results

27 individuals (m = 21/f = 6) met inclusion criteria and were examined. 59 % of all patients received their pump implantation in our clinic, 41 % were operated externally. 96 % of all implanted pumps were battery operated and from the company Medtronic (Synchromed series I and II). Only one patient was supplied with a gas-powered Medstream pump. The average operating time of the battery-powered pumps was 72 months.

25 patients had spasticity due to a spinal cord injury, while 2 patients suffered from multiple sclerosis. 80 % of the SCI population were classified AIS A. AIS B and C were each set at 8 %, while AIS D was set at 4 %, as shown in Table 2**.** The main cause for paralysis was traffic accident (n=14/56 %), stairs overthrow (n=4/16 %), spinal stenosis (n=2/8 %), spondylodiscitis (n=1/4 %), falls from a horse (n=2/8 %) and multiple sclerosis (n=2/8 %). The level of paralysis was 55 % cervical and 45 % thoracic. The median population age at implantation of a pump was 46 years (range: 24–76 years). The median onset of paralysis was 12 months (3–71month) previous **(**Table 1**).** For patients with multiple sclerosis, the time of onset was chosen based on when neurological symptoms (paralysis and/or spasticity) were mentioned or diagnosed in the medical records. The time point was defined similarly for non-traumatic spinal cord injuries, since in some cases, the onset of paralysis could not be spontaneously determined at a specific time. However, since the duration from the onset of the disease to the date of pump implantation is given in months, we believe a certain time span can be accepted in this regard.

**Table 1:** Demographic characteristics.

**Variable**	**Study group** (n = 27)
*Sex*	
Male	21 (78 %)
Female	6 (22 %)
*Mean age at first pump implantation*	46 years (24–79 years)
*Mean time of implantation after SCI-onset*	12 months (3–71month)

**Table 2:** Spinal cord injury characteristics.

**Variable**	**Study group** (n = 27)
*SCI cause*	
falls from horse	02 (08 %)
Infection	01 (04 %)
spinal stenosis	02 (08 %)
traffic accidents	14 (56 %)
stair falls	04 (16 %)
multiple sclerosis	02 (08 %)

*AIS Grade*	
A	21 (80 %)
B	02 (08 %)
C	02 (08 %)
D	01 (04 %)
*Neurological Level of Injury*	
cervical (C1-C7)	11 (35 %)
thoracic (Th1-Th12)	18 (56 %)

The Indication was made for all patients with AS IV°. With intrathecal therapy, an improvement to AS I° in 40 % and II° in 60 % was achieved**.** 31 % of all cases had previously been treated unsuccessfully with a triple combination of oral antispasmodics, 54 % with double therapy, and 15 % with monotherapy. In 42 % of cases, a test dose of 50 μg intrathecal baclofen was sufficient to indicate pump implantation, regardless of the number of antispasmodics previously administered. The mean test dose for monotherapy was 66.67 ± 11.78 μg, for dual therapy 100 ± 41.23 μg, and for triple therapy 81.25 ± 47.97 μg (Table 3). Retrospectively, the reasons for choosing a test dose could not be fully reconstructed in all cases, as they were not consistently documented and, therefore, were not further investigated or included in the analysis.

**Table 3:** Start and effective dose of intrathecal Baclofen.

	Testing Dose (μg/d)	Starting Dose (μg/d)	Effective Dose (μg/d)	Hospital Stay (days)
Monotherapy				
Mean ± SD	66.67 ± 11.78	110 ± 14.4	**250 ± 15**	28.3 ± 11.7
*Min – Max*	*50–75*	*100–120*	*100–410*	*18–45*
**Dual Therapy**				
Mean ± SD	100 ± 41.23	181.1 ± 104.8	**388.4 ± 120.1**	54.2 ± 46.4
*Min – Max*	*50–150*	*110–400*	*271–600*	*11–118*
**Triple Therapy**				
Mean ± SD	81.25 ± 47.97	162 ± 43	**417.3 ± 118.3**	41.5 ± 42.4
*Min – Max*	*50–150*	*96–200*	*240–520*	*14–108*

Patients who received spasmolytic monotherapy prior to pump implantation required a starting intrathecal dose of 110 μg a day and an increase in the average baclofen dose to 250 μg a day until a satisfactory level of spasticity was achieved, typically within an average hospital stay of 28.3 ± 11.7 days, during which the effective dose was determined. Patients with dual therapy started at an average of 181.1 μg a day and required an increase in the dose to around 388.4 μg a day, with the effective dose being assessed over an average hospital stay of 54.2 ± 46.4 days. Those initially on the oral triple combination began at 162 μg a day and received an increase in baclofen daily rate to approximately 417.3 μg a day over time, with the effective dose evaluated over an average stay of 41.5 ± 42.4 days. The mean increase in baclofen daily flow rate from implantation until a satisfactory clinical effect was achieved was approximately 180 μg per day, usually within these timeframes.

All dose increases were carried out in a hospital in-patient setting and were performed individually according to the clinical findings of spasticity and the patient's condition, without a fixed schedule or fixed time intervals. In clinical routine, however, for reasons of patient observation and safety, the increases were usually not made on weekends or holidays. Dose adjustments were generally made at the beginning and middle to end of the week, and a 2-3-day observation period was usually waited before making another increase. The increases in the run rate usually amounted to no more than 10 % per increase step, with the exception of the first 2–3 increases after pump implantation, as the run rates are still relatively low overall. The starting dose was also determined based on the clinical experience of the investigators and the clinical situation of the patient.

In 75 % of all patients, no complications occurred throughout the intrathecal treatment period. Complications occurred in 25 % (n = 7), comprising 3 infections, 2 skin perforations, 1 catheter occlusion, and 1 pump malfunction. All skin perforations were located abdominally, mainly along the skin suture of the pump pocket and were successfully managed with soft tissue revision and pump replacement, allowing continued use of the pump. Two early infections, occurring 2–3 weeks post-implantation, necessitated pump explantation in both cases. In one instance, the pump was re-implanted during the same surgical procedure following soft tissue revision, whereas in the other, re-implantation was deferred due to the severity of the infection, and the patient subsequently declined further implantation, due to concerns about further complications. One case involved an undetermined infection with high fever and no identifiable focus; pump removal was performed for infection management, with intraoperative samples showing no signs of infected pump material. Following stabilization and resolution of the fever, re-implantation occurred after six months, with the pump functioning normally thereafter. In one case, catheter tip occlusion, likely due to a material defect, led to pump removal and re-implantation during the same surgical procedure, after which normal pump function was resumed. A pump malfunction occurred following battery depletion, with failure to restart the motor, necessitating pump replacement. Eighty-five percent of patients experiencing complications were able to continue intrathecal treatment following revision. Complications were observed in 25 % of cases (n=3/7 infection, n=2/7 skin perforation, n=1/7 catheter occlusion, n=1/7 pump malfunction). 85 % of all patients who suffered a complication could be further treated intrathecally after revision.

## 4 Discussion

Spinal spasticity is often a functionally disabling complication of upper motor neuron damage. Treatment with classic oral antispasmodics frequently leads to challenges, including therapy discontinuation due to central adverse effects. Intrathecal baclofen therapy (ITB) represents a viable and effective alternative in well-selected patients and individual cases. However, due to the potential toxicity of baclofen during intrathecal administration and fluctuations in its effects during intrathecal screening or dose titration at the initiation of continuous treatment post-implantation, clinicians must exercise particular caution. Careful management is essential to minimize risks and ensure patient safety.

Our study evaluated on determining the relationship between prior oral antispasmodic therapy (monotherapy or combination therapy) and the required intrathecal baclofen effective dose. As part of the intrathecal test dose episode, Romito et al. described that the ITB screening test commonly starts with a baclofen bolus dose of 50 μg followed by observation for 8 h. If no response is identified, a subsequent ITB bolus dose of 75 μg is administered at least 24 h after the initial 50 μg bolus. The patient is again monitored for a positive response for 8 h. If this dose is not adequate to provide a positive response, a final screening bolus can be administered with a maximum ITB dose of 100 μg after 24 h (Romito et al., 2021; Boster et al., 2016). In our observation, a initial dose of 50 μg intrathecal baclofen was sufficient in 42 % of all cases to indicate pump implantation. Only 34 % required even more than 100 μg.

This finding is consistent with the 2024 guidelines (Platz et al., 2024)., which recommend an initial bolus of 50 μg, with possible increases to 75 μg or 100 μg, depending on patient response. However, the guidelines also emphasize that the decision to proceed with ITB therapy should only be made after insufficient response to physical therapy, physiotherapy, and oral medication in cases of generalized or multisegmental spasticity (Motta et al., 2007).**,** With the aim of minimizing lumbar punctures prior to the indication for pump-assisted baclofen treatment, no useable trend could be identified from the available data with which the optimal level of the intrathecal starting dose could be more ideally determined. Additionally, we examined the average initial dose at the start of continuous ITB therapy and the effective target dose required to achieve satisfactory spasticity control promptly and safely.

Skoog et al. found, that SCI patients needed a median daily dose of 260 μg/24 h (range, 30–725 μg) one year after the start of ITB. MS patients required a lower dose, with a median dosage of 112 μg/24 h (range, 66–420 μg). The mean dosage for the SCI patients was 257 μg/24 h (189–325) and for the MS patients 158 μg/24 h (95 % CI, 113–204), and the difference was significant (*t*-test p = 0.02) (Skoog and Hedman, 2019).In contrast, in our study, mean starting doses for intrathecal baclofen were 180 μg a day and the effective target dose was 360 μg/day. The initial daily baclofen starting dose rate varied from 110 μg (for patients with monotherapy) to 181 μg (for patients with dual therapy) and 162 μg a day (for patients with triple therapy) depending on the previous oral pretreatment and the differentiation between monotherapy and combination therapy. In our analysis, the fact that those patients who had previously been treated with 3 oral drugs received a lower starting dose of baclofen is due to the fact that the patients with dual pretreatment had reduced and discontinued almost all oral antispasmodics prior to pump implantation. Those who received a triple combination were usually treated with an overlapping further oral antispasmodic (e.g. clonazepam or tizanidine) at the beginning of the intrathecal baclofen therapy before the last oral drug could finally be discontinued after the effective baclofen target dose had been reached. Based on this knowledge, we are now trying to reach the required target dose more quickly and safely and on the other hand, to switch from oral therapy to intrathecal application with as little clinical fluctuation as possible.

McIntyre et al. reported an overall improvement of spasticity, assessed with AS, followed by ITB therapy (McIntyre et al., 2014). Coffey et al. demonstrated a decrease in Ashworth scores from 3.9 before to 1.7 after implantation (Coffey et al., 1993). Azouvi et al. reported a statistically significant decrease in Ashworth scores at 6 months post-treatment (Z = −3.79; P < 0.001) (Azouvi et al., 1996). During an initial intrathecal baclofen bolus treatment for subjects, Abel and Smith reported that AS decreased from 3.8 before the bolus to 1.5 after. Although no further statistical analysis was conducted, the authors reported that AS remained reduced to ≤2 points even after pump implantation in all but three subjects (Abel and Smith, 1994). Loubster et al. reported that AS scores decreased from 3.79 ± 0.69 before to 2.00 ± 0.96 after implantation (P < 0.001) (Loubster et al., 1991). In our study, all patients suffered from generalized spasticity AS IV° before starting the intrathecal baclofen therapy. After ITB pump implantation and reaching the effective daily dose of baclofen, an improvement of spasticity to AS I° in 40 % and to AS II° in 60 % of all cases was achieved.

Saval et. al. reported an overall complication prevalence of 16 % over 3 years in a retrospective study with 57 individuals. Complications were reported in 2 individuals with catheter migration, 6 with fractured catheters, and 1 with a pump infection with meningitis, with subsequent catheter removal and replacement (Saval and Chiodo, 2010). In our study we observed complications in 25 % of cases (3 infection, 2 skin perforation, 1 catheter occlusion, 1 pump malfunction). 85 % of all patients who suffered a complication could be further treated intrathecally after revision. However, it is important to emphasize that these differences cannot be considered statistically significant without a formal statistical analysis of the case numbers and complication types.

### 4.1 Limitations of this study

Caution is warranted in interpreting the findings of this analysis due to the relatively small sample size. Additionally, some data were sourced from medical reports provided by external hospitals, which may introduce variability. Another concern is the inclusion of two multiple sclerosis (MS) patients in the study group. While the inclusion of these patients may have been necessary to increase the sample size, it is important to note that MS patients generally require lower baclofen doses compared to patients with other conditions, which could reduce the homogeneity of the group. This inclusion may have influenced the overall outcomes of the study. Therefore, this limitation should be considered when interpreting the findings.

Furthermore, a limitation of the study lies in the retrospective nature of the data collection, where the exact day on which the effective baclofen dose was determined was not always clearly documented in the medical records, although a clear clinical improvement in spasticity was observed during this period. To improve comparability, we used the number of hospital days until discharge as a proxy for the time period during which the effective dose was assessed. However, given the small sample size, this approach may not fully capture the exact timing or significant differences in dose titration. To draw more robust and meaningful conclusions about intrathecal baclofen treatment, further research involving larger patient cohorts and prospective study designs is essential.

## 5 Conclusion

In selected cases, intrathecal baclofen therapy serves as an effective alternative for patients who have not achieved satisfactory outcomes with oral baclofen treatment. Improved understanding of the necessary screening doses, based on previous antispasmodic therapy and spasticity severity, could enable more precise selection of test doses and also reduce the frequency of cerebrospinal fluid punctures. Establishing the expected starting and effective target doses facilitates a more efficient, targeted, and safe transition from oral to intrathecal therapy and minimizes or avoids undesirable and potentially harmful fluctuations in therapeutic effects during rotation. Our study suggests a trend indicating that a higher number of spasmolytic medications used correlates with a higher effective baclofen dose required to achieve clinical improvement. Despite the relatively high complication rate observed in our study population, the majority of patients achieved satisfactory outcomes with intrathecal baclofen following appropriate management of complications.

Given the limitations of our retrospective study, we will continue to follow the Romita regimen, as our current data set, due to its small sample size, does not justify any fundamental deviations. However, moving forward, we aim to implement a more targeted approach to dose escalation by better estimating the likely target dose based on prior medication use. Our goal is to develop an individualized and risk-adapted titration plan that systematically records both absolute and percentage-based dose adjustments and clearly documents clinical assessments regarding spasticity reduction and baclofen tolerability during the titration phase. This approach is intended to enable efficient completion of the titration process in the shortest possible time, while ensuring maximum patient safety and improved procedural transparency. Nonetheless, the observed trends may provide a useful basis for planning a prospective, statistically more robust study with a larger cohort.

## Data available statement

The datasets generated and analyzed during the current study are available from the corresponding author on reasonable request.

## Ethical approval

The study has received approval from the Ethics Committee of the State Medical Association of Hesse, with the reference number 2024-3838-evBO.

## Declaration of competing interest

The authors declare that they have no known competing financial interests or personal relationships that could have appeared to influence the work reported in this paper.
